# Clinical features and outcome of multiple primary malignancies involving hepatocellular carcinoma: A long-term follow-up study

**DOI:** 10.1186/1471-2407-12-148

**Published:** 2012-04-17

**Authors:** Qing-An Zeng, Jiliang Qiu, Ruhai Zou, Yijie Li, Shengping Li, Binkui Li, Pinzhu Huang, Jian Hong, Yun Zheng, Xiangming Lao, Yunfei Yuan

**Affiliations:** 1 State Key Laboratory of Oncology in South China/Department of Hepatobiliary Oncology, Sun Yat-Sen University Cancer Center, 651 Dongfeng Road East, Guangzhou, Guangdong, 510060, China; 2 Department of General Surgery, the Fifth Affiliated Hospital, Sun Yat-Sen University, Zhuhai, China

**Keywords:** Neoplasm, Multiple primary, Extrahepatic, Hepatocellular carcinoma, Prognosis

## Abstract

**Background:**

The prolonged survival of individuals diagnosed with cancer has led to an increase in the number of secondary primary malignancies. We undertook to perform a definitive study to characterize and predict prognosis of multiple primary malignancies (MPM) involving hepatocellular carcinoma (HCC), due to the scarcity of such reports.

**Methods:**

Clinicopathological data were analyzed for 68 MPM patients involving HCC, with 35 (target group) underwent curative liver resection. Additional 140 HCC-alone patients with hepatectomy were selected randomly during the same period as the control group.

**Results:**

Of the 68 patients with extrahepatic primary malignancies (EHPM), 22 were diagnosed synchronously with HCC, and 46 metachronously. The most frequent EHPM was nasophargeal carcinoma, followed by colorectal and lung cancer. Univariate analysis demonstrated that synchronous (*P* = 0.008) and non-radical treatment for EHPM (*P* < 0.001) were significant risk factors associated with poorer overall survival (OS). While, Cox modeling revealed that the treatment modality for EHPM, but not the synchronous/metachronous determinant, was an independent factor for OS, and that therapeutic option for HCC was an independent factor for HCC-specific OS. Moreover, no HCC-specific overall and recurrence-free survival benefit were observed in the control group when compared with that of the target group (*P* = 0.607, *P* = 0.131, respectively).

**Conclusions:**

Curative treatment is an independent predictive factor for OS and HCC-specific OS, and should been taken into account both for synchronous and metachronous patients. MPM patients involving HCC should not be excluded from radical resection for HCC.

## Background

Hepatocellular carcinoma (HCC) ranks as the fifth most common tumor and third leading cause of global cancer-related mortality [1]. The improvement in early diagnosis and the advances in treatment, including new targeted therapy, result to greatly improve the prognosis of HCC patients. Consequently, there is an increasing need to consider the development of multiple primary malignancies (MPM), which may influence the treatment for HCC.

Although the incidence of MPM has begun to rise during the past decade, knowledge of the clinical features and outcomes of MPM remains limited. From a clinical viewpoint, it is important to determine whether cancer-survivors are predisposed to suffer some specific tumor types, and whether the prognosis of MPM patients including HCC differ from those afflicted with liver-limited malignancy in term of HCC-specific survival. Moreover, subsequent malignancies may not only be attributable to prior cancer-related treatment, but may also mirror the impact of therapy-related factors, shared etiologies, host immune responses, environmental exposures, and combination effects of the gene-environment. Clearly, as the treatment for the first cancer might limit therapeutic potential upon diagnosis of a secondary cancer, more information regarding the MPM patients with HCC is needed to facilitate the establishment of therapeutic strategies for those patients.

To the best of our knowledge, only two papers examined the clinicopathological factors of MPM with more than 20 patients [2,3], who had received radical hepatectomy for primary HCC, have been published within the last 20 years. Although small sample-sized studies were performed in Japan [4,5], Taiwan [6], and Western countries [7], none of them were from Mainland China, an area with a high incidence of HCC (> 20 annual cases per 10^5^ people) [8]. This retrospective study was designed to characterize MPM patients, to explore long-term prognosis, and to investigate the impact of extrahepatic primary malignancy (EHPM) on survival of HCC in China.

## Methods

### Definition of second primary malignancy

A second primary neoplasm was defined according to IARC (International Agency for Research on Cancer) rules [9,10], as follows: 1. The existence of two or more primary cancers does not depend on time; 2. Both tumors are confined to primary sites, and no direct connections between the tumors exist; 3. One tumor should only be recognized in an organ or a pair of organs or tissue (as defined by the code of the International Classification of Diseases (ICD)); 4. Rule 3 does not apply if tumors in an organ are of a different histology; 5. Be different in histological type when diagnosis of pathology available.

### Patients

Between Jan. 1989 and Apr. 2008, 68 patients experienced MPM involving HCC in the Sun Yat-Sen University Cancer Center, and a total of 2841 HCC-alone patients received hepatectomy during the same period. Among the 68 MPM patients, 35 (51.5%) underwent radical hepatectomy and were defined as the target group. To investigate the influence of EHPM on HCC-specific survival, 140 surgical HCC-alone patients (four times of the target group) were randomly selected from 2841 patients as the control group. The definitions for synchronous and metachronous (interval > 6 months) have also been addressed in several previous studies [11-13]. HCC was noninvasively diagnosed on the basis of the typical radiological features on ultrasonography, computed tomography (CT), magnetic resonance imaging (MRI), and a combination of alpha-fetoprotein (AFP) levels [14]. Positron emission tomography scan with ^18^ F-fluorodeoxyglucose was carried out since Oct 2005 in our center, which was performed if a secondary primary cancer was suspected, and 16 patients were found using this method. Of note, histopathology from biopsy or liver resection remains the golden diagnostic standard. Patients who met the diagnosis criteria of autoimmune hepatitis [15] or cholangiocarcinoma were excluded. All the recruited patients gave written informed consent before examination and treatment. The study protocol was approved by the Ethics Committee of Sun Yat-Sen University Cancer Center and conformed to the ethical guidelines of the Helsinki Declaration.

Overall survival (OS) was calculated between the date of the first primary treatment and date of death or last follow-up of either the first or the subsequent malignancy. HCC-specific OS was defined as the interval from the date of diagnosis of HCC to the patient’s death of related HCC, or last follow-up, whileas HCC-specific recurrence-free survival (RFS) was defined as the interval from the date of diagnosis of HCC to the tumor recurrence or last contact with the patient if recurrence did not occur. Curative therapy was defined as treatment for the EHPM with intent to cure according to different tumor classification, e.g., surgery for colorectal, gastric, lung, esophagus, cervical, breast, thyroid, oral, and renal cancer, or radiochemotherapy for nasopharyngeal cancer and non-Hodgkin lymphoma. Supportive therapy was defined a treatment that encompassed nonspecific therapeutic factors for tumor, such as facilitating affect expression. And the palliative treatment was an intermediate one between them.

### Statistical analysis

Descriptive statistics are expressed as mean ± standard deviation. The *T*-test, chi-square test or Fisher’s exact test, where appropriate, were used for univariate comparisons. For univariate survival analysis, plots were generated using the Kaplan-Meier method. A Multivariate model was built to evaluate the risk associated with prognostic parameters. Differences were considered significant when *P* < 0.05. All statistical analyses were performed using SPSS version 16.0 statistical software package (SPSS, Inc, Chicago, IL). The study was censored on Jul 30, 2011.

## Results

### Patient characteristic

Of the 68 patients, 67 (98.5%) had double cancers and 1 (1.5%) suffered from triple cancers. The most frequent site preceding or following HCC was nasopharynx (11/68, 16.2%), followed by colorectal (10/68, 14.7%), lung (6/68, 8.8%), skin, gastric, esophagus, cervical, breast, thyroid, oral, urinary bladder, and renal cancer as well as non-Hodgkin lymphoma. The different tumor types and therapeutic option for EHPM are presented in Table 1. Of the 68 patients with EHPMs, 22 were diagnosed synchronous with HCC, and 46 belonged to the metachronous group. To stratify the metachronous group clearly, 35 patients occurred prior to HCC (prior group), and 11 posterior to HCC (post group). In addition, 18 patients had HCC diagnosed before EHPM, 12 patients had HCC and EHPM during the same hospital admission, and the remaining 38 were diagnosed with EHPM occurring ahead of HCC, with 19 patients underwent hepatectomy.

**Table 1:** Locations of extrahepatic primary cancers implicating hepatocellular carcinoma

**Site of extrahepatic** **primary cancer**	**Synchronous group (n = 22)**	**Metachronous group (n = 46)**	**Total** **(n = 68)**
Head & neck	8 (36.4%)	15 (32.6%)	23 (33.8%)
nasopharynx	2 (1^R^ 1^S^) *	9 (9^R^)	11
tonsil	1 (1^C^)	0	1
larynx	1 (1^C^)	0	1
vocal cord	1 (1^S^)	1 (1^O^)	2
mouth	0	1 (1^O^)	1
tongue	2 (1^C^1^S^)	1 (1^O^)	3
gingiva	0	1 (1^O^)	1
soft palate	1 (1^S^)	0	1
thyroid gland	0	2 (2^O^)	2
Digestive system	8 (36.4%)	10 (21.7%)	18 (26.5%)
esophagus	1 (1^S^)	4 (3^O^1^C^)	5
stomach	2 (2^O^)	1 (1^O^)	3
colorectal	5 (5^O^)	5 (5^O^)	10
Urogenital system	1 (4.5%)	5 (10.9%)	6 (8.8%)
prostate	0	1 (1^O^)	1
urinary bladder	1 (1^O^)	1 (1^O^)	2
kidney	0	2 (1^O^1^C^)	2
ovary	0	1 (1^O^)	1
Others	5 (22.7%)	16 (34.8%)	20 (29.4%)
lung	2 (1^C^1^S^)	4 (1^O^2^C^ 1^R^)	6
breast	0	4 (4^O^)	4
skin	1 (1^O^)	4 (4^O^)	5
Non Hodgkin’s lymphoma	1 (1^R^) ^†^	3 (3^C^)	3
melanoma	0	1 (1^O^)	1
Soft tissue tumor	1 (1^O^)	0	0

* number^treatment^ Treatment include: O operation; R radiotherapy; C chemotherapy; S supportive treatment.

^†^ The patient suffered the third primary malignancy, acute monocytic leukemia.

**Figure 1:**
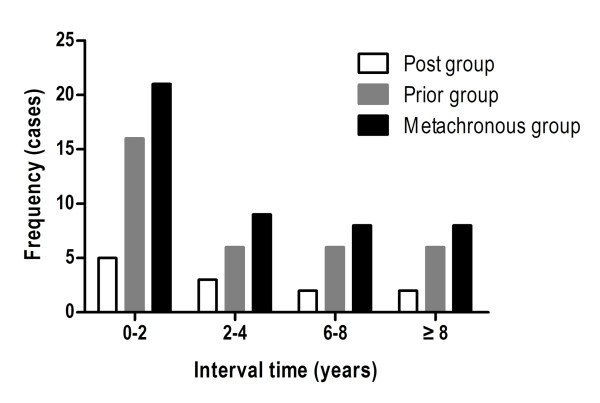
Schematic graph showing frequency distribution of follow-up time after diagnosis of the first primary tumor in the prior, post and metachronous groups.

In the entire study, 61 cancer patients were male and 7 were female. Hepatitis B surface antigen (HBsAg) was detected in 66.2% (45/68) patients, while hepatitis C antibody was negative in any of the patients. Cirrhosis was present in 75.0% (51/68) of the cases. HCC patients were diagnosed through the following routes: 35 were diagnosed on the basis of histology via hepatectomy, 30 by standard clinical and imaging criteria combined with AFP levels, and 3 cases underwent biopsies following an uncertain clinical diagnosis. As demonstrated in Figure 1, 45.7% (21/46) of the metachronous patients experienced their secondary cancer within 2 years of the initial cancer diagnosis, while 55.2% (30/46) of the patients were diagnosed within 5 years, with a clear predominance in the prior group. The diagnostic intervals between the two cancers ranged from 6.5 months to 14.8 years in the metachronous patients (51.0 ± 47.4 months). No significant difference was observed in the interval time between the post and prior groups (51.3 ± 50.4 vs. 50.2 ± 37.8, *P* = 0.946).

The median follow-up time was 7.8 years, with a range of 3.2 to 16.1 years. During the follow-up, 32 (47.1%) patients were still alive, while 29 (42.6%) patients died of HCC-related causes, 4 (5.9%) of EHPM-related causes, and 3 (4.4%) of unclear causes.

Of the 68 HCC patients with a history of EHPM, 35 underwent liver resection, 7 local ablation considering severe cirrhosis, 14 tranarterial chemoembolization, and 11 supportive treatment. Moreover, of the 68 EHPM patients, 54 received curative therapy for EHPM, 8 palliative therapy, and 6 supportive treatment (Table 2). No significant difference regarding gender, age, Child-Pugh grading, AFP, GGT level, TNM stage, and treatment for HCC were observed in the synchronous and metachronous groups (Table 2). Interestingly, the rate of patients received radical treatment for EHPM in the metachronous group was higher than that of the synchronous group (*P* < 0.001, Table 2).

**Table 2:** Patient demographical characteristics of 68 multiple primary patients with hepatocellular carcinoma

**Characteristic**	**Total**	**Synchronous group (n = 22)**	**Metachronous group (n = 46)**	*P *value *
Age (years) ^†^				
< 58 ^b^	33	8	25	0.165
≥ 58	35	14	21	
Gender				0.514
Male	61	21	40	
Female	7	1	6	
HBsAg status				0.393
Negative	23	9	14	
Positive	45	13	32	
Serum AFP level (ng/ml)				0.085
< 25	30	13	17	
≥ 25	38	9	29	
Serum GGT level (U/ml)				0.181
< 50	23	5	18	
≥ 50	45	17	28	
Child-Pugh classification				0.932
A	49	16	33	
B	19	6	13	
TNM stage				0.735
I	43	14	29	
II	9	2	7	
III	16	6	10	
Treatment for HCC				0.309
Resection	35	9	26	
Ablation	7	3	4	
TACE	14	4	10	
Supportive	11	6	5	
Treatment for extrahepatic cancer				<0.001
Curative	54	12	42	
Palliative	8	4	4	
Supportive	6	6	0	

HCC hepatocellular carcinoma, HbsAg Hepatitis B surface Antigen, AFP α-fetoprotein, GGT gamma-glutamyltransferase, TNM tumor-node-metastasis, TACE transarterial chemoembolization.

* Chi-square or Fisher’s exact test.

^†^ Patients were divided according to the median age.

### Univarite and multivariate analysis of prognosis

**Figure 2:**
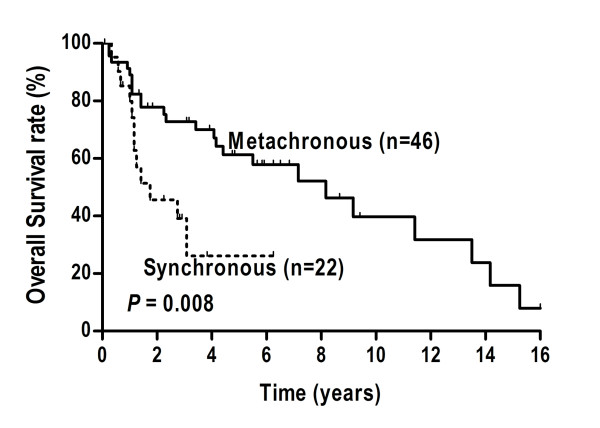
Comparison of Kaplan-Meier curves for overall survival between the synchronous and the metachronous groups.

The actuarial 1-, 3-, and 5-year OS rate for 68 MPM patients were 89.3%, 63.0%, and 51.8%, respectively. The 1-, 3-, and 5-year HCC-specific OS rate for the 35 surgical patients were 75.1%, 46.3%, and 35.9%, respectively. The difference reached significance between the metachronous and synchronous patients in OS (*P* = 0.008, Figure 2), but not in HCC-specific OS (*P* = 0.360, Table 3).

**Table 3:** Univariate analysis of prognostic factors in term of overall survival and HCC-specific overall survival

**Characteristic**	**HCC-specific overall survival rate (%)**			**Overall survival rate (%)**		
	**3y**	**5y**	***P *value**	**3y**	**5y**	***P *value**
Gender			0.901			0.815
Female (*n* = 7)	45.5	38.4		85.7	64.3	
Male (*n* = 61)	51.4	34.3		58.0	50.4	
Age (years)*			0.441			0.612
< 58 (*n* = 33)	52.3	47.1		62.7	62.7	
≥58 (*n* = 35)	40.5	27.1		59.8	41.7	
HBsAg status			0.148			0.909
Negative (*n* = 23)	40.0	28.6		56.0	49.1	
Positive (*n* = 45)	50.8	36.2		62.4	51.8	
AFP (ug/l)			0.034			0.252
≤ 25 (*n* = 30)	62.9	48.4		68.9	68.9	
>25 (*n* = 38)	32.6	27.2		54.9	39.6	
GGT level (u/l)			0.040			0.073
≤ 50 (*n* = 23)	71.8	71.8		76.5	76.5	
> 50 (*n* = 45)	35.9	29.4		52.9	43.8	
Cirrhosis			.255			0.078
No (*n* = 17)	65.8	65.8		85.6	66.3	
Yes (*n* = 51)	40.9	29.5		52.9	46.8	
Child-Pugh classification			0.334			0.083
A (*n* = 49)	50.8	39.7		64.6	59.8	
B (*n* = 19)	36.4	27.3		48.2	32.8	
Tumor size (cm)			0.865			0.537
≤5 (*n* = 28)	49.0	42.0		62.8	47.3	
>5 (*n* = 40)	44.6	31.5		59.7	48.0	
Multiple tumors			0.001			0.221
No (*n* = 49)	58.0	48.1		65.3	57.7	
Yes (*n* = 19)	11.1	11.1		62.1	41.4	
Vascular invasion			0.001			0.977
No (*n* = 58)	51.7	40.2		60.2	52.3	
Yes (*n* = 10)	0	0		66.8	53.4	
TNM stage			<0.001			0.698
I (*n* = 43)	58.4	50.0		61.4	54.0	
II/III (*n* = 25)	17.1	0		60.1	47.7	
Time of appearance			0.360			0.008
Synchronous (*n* = 22)	34.5	23.0		25.7	25.7	
Metachronous (*n* = 46)	50.3	40.3		72.8	61.3	
Treatment for HCC			<0.001^†^			0.160
Resection (*n* = 35)	71.9	52.0		64.9	64.9	
Ablation (*n* = 8)	50.8	50.8		66.7	50.0	
TACE (*n* = 14)	0	0		50.0	37.6	
Supportive (*n* = 11)	0	0		40.0	26.7	
Treatment for EHPM			0.067			<0.001^‡^
Curative (*n* = 54)	47.3	33.2		72.4	63.4	
Palliative (*n* = 8)	19.2	19.2		35.7	17.9	
Supportive (*n* = 6)	11.7	11.7		0	0	

HCC: hepatocellular carcinoma, HBsAg: hepatitis B surface antigen, AFP: α-fetoprotein, GGT: gamma-glutamyltransferase, TACE: transarterial chemoembolization, EHPM: extrahepatic primary malignancy.

* Patients were divided according to the median age.

^†^ Resection vs. Ablation *P* = 0.346; Ablation vs. TACE *P* < 0.001; TACE vs. Supportive *P* = 0.413.

^‡^ Radical vs. Palliative *P* < 0.001; Palliative vs. Supportive *P* = 0.122.

The effects of clinical variables on the outcome were evaluated. Treatment options for HCC (resection), level of GGT (≤ 50 U/L) and AFP (≤ 25 ug/L), tumor number (solitary), vascular invasion (absent), and TNM stage (TNM stage I) were significant factors associated with favorable HCC-specific OS in univariate analysis (Table 3). By multivariate analysis, only treatment for HCC remained an independent predictor of HCC-specific OS (Table 4). Likewise, time of appearance (synchronous/metachronous) and treatment for EHPM were strong and significant predictors of OS in univariate analysis (Table 3). Finally, Cox analysis revealed the treatment for EHPM was the independent factor in OS (Table 4).

**Table 4:** Multivariate analysis of prognostic factors in term of overall survival and HCC-specific survival

**Characteristic**	**HCC-specific overall survival**		**Overall survival**	
	***P *value**	**HR (95.0% CI)**	***P *value**	**HR (95.0% CI)**
AFP level	0.610	1.257 (0.522-3.030)	-	-
GGT level	0.449	1.485 (0.534-4.130)	-	-
Tumor number	0.174	2.151 (0.713-6.491)	-	-
Vascular invasion	0.148	2.550 (0.717-9.064)	-	-
TNM stage	0.250	2.016 (0.611-6.656)	-	-
Treatment for HCC	0.001	1.893 (1.297-2.764)	-	-
Time of appearance	-	-	0.535	1.329 (0.542-3.261)
Treatment for EHPM	-	-	<0.001	2.758 (1.581-4.811)

HCC: hepatocellular carcinoma, AFP: α-fetoprotein, GGT: gamma-glutamyltransferase, TNM: tumor-node-metastasis, EHPM: extrahepatic primary malignancy, HR: Hazard ratio, CI: confidence interval.

### Impact of EHPM on HCC-specific survival

**Figure 3:**
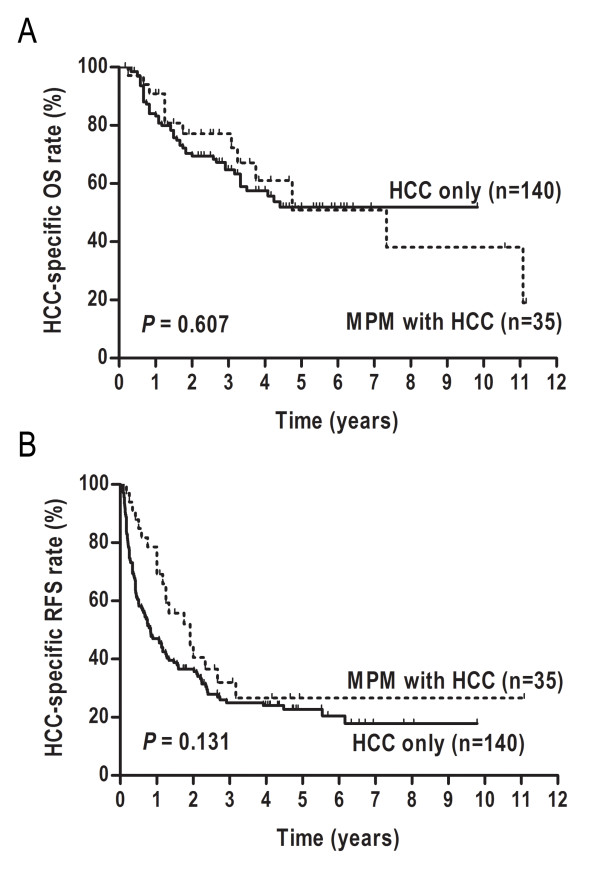
Comparison of Kaplan-Meier curves between the 140 HCC-alone patients and 35 multiple primary malignancies (MPM) patients with surgical HCC in term of HCC-specific overall survival (A), and HCC-specific recurrence-free survival (B).

To investigate the impact of EHPM on survival of HCC, 140 HCC-alone randomly selected patients were included to compare with the 35 resected HCC patients with EHPM. No significant differences were found between the two groups in clinicopathological features (Table 5). Moreover, the postoperative complications were similar in two groups (*P* = 0.373), which included hemorrhage, liver failure, biliary fistula, would infection, chest infection, and cardiac arrhythmia. There was no postoperative death occurred within 30 days after resection in both groups. Median HCC-specific OS for the control, and target group were 2.54 years, and 2.56 years, respectively. There were no significant HCC-specific OS and RFS difference between the two groups (*P* = 0.607, *P* = 0.131, respectively, Figure 3).

**Table 5:** Compared clinicalpathological features between 35 surgical HCC patients with multiple primary tumor and 140 HCC patients without extrahepatic tumor

**Characteristic**	**Multiple primary** **tumor group (n=35)**	**Control group** **(HCC-alone) (n=140)**	***P *value**^**†**^
Age (years) *	56.3±12.6	59.1±11.2	0.317 ^‡^
Gender (Female: Male)	5:30	30:115	0.616
HBsAg (Negative: Positive)	7:28	12:128	0.101
AFP level (<25: ≥25) (ng/ml)	17:18	44:96	0.057
GGT level (<50: ≥50) (U/ml)	11:24	53:87	0.480
Child-Pugh classification (A:B)	32:3	134:6	0.549
Tumor size (<5cm : ≥5cm)	12:23	40:100	0.508
Tumor number (Solitary: Multiply)	30:5	132:8	0.171
Vascular invasion (No: Yes)	32:3	131:9	0.940
Tumor capsule (No: Yes)	19:16	60:80	0.224
Cirrhosis (No: Yes)	8:27	23:117	0.373
Surgical margin (<2cm : ≥2cm)	20:15	57:83	0.080
TNM stage (I: II: III)	26:4:5	112:18:10	0.401
Complication (No: Yes)	6:29	14:126	0.373

HCC: hepatocellular carcinoma, HBsAg: hepatitis B surface antigen, AFP: α-fetoprotein, GGT: gamma-glutamyltransferase, TNM: tumor-node-metastasis.

* Mean ± Standard Deviation.

^†^ Chi-square test for difference between proportions (except that of Age).

^‡^*T* test for difference between means.

## Discussion

The last decade has experienced a steady increase in the incidence of MPM [16]. With the expected survival of oncology patients prolonged, it is no longer rare to observe a subsequent primary malignancy in long-term follow-up, especially those from therapy-related carcinogenesis [17,18]. A recently published paper provided by the Surveillance, Epidemiology and End Results Program estimated 7.9% of cancer-survivors were living with a history of more than one primary malignancy in the U. S [16]. The reported prevalence estimated that cancer-survivors were at an increased risk of being diagnosed with a secondary primary malignancy [19-21]. Bhatia et al. showed the risk of second tumor was increased approximately 18 times in survivors of childhood Hodgkin’s lymphoma [21]. The incidence of second neoplasm in cancer-survivors (2.3%, 68/2909) of our center was in accordance with previous literature [6,16,22,23], which was higher than that of the normal population [1]. We hypothesized that the improvement in early screening technologies, the advent of new molecular targeted agents, and elevated risk of carcinogenesis during the anti-cancer therapy yielded an increase prevalence of second cancer in the individuals ever diagnosed malignancy.

Several studies have demonstrated that the risk factors for poor survival of MPM patients, including male, elderly, and tumor stage [11,24]. While, our study showed no statistical difference for OS was observed in conventional clinicopathological parameters, which was agreed with other studies [5,23]. This discrepancy may be due to the different disease etiologies and heterogeneity of study populations. We believe that a study of patients from a large, multicenter, and multigeographic area would reach conclusions more powerful.

A unimodal distribution of interval time among the prior and post subgroups of the metachronous group was shown in Figure 1. Based on the time since the first cancer diagnosis, the overall risk was decreased following the first 2 years. Considering that most secondary cancers were detected by periodic examination, a postoperative periodic checkup should be performed intensively both for the prior and post groups, especially during the first 2 years. Interestingly, we found that an obvious predominance exhibited in the prior group. We hypothesized that the poor prognosis of HCC may explain partially to this circumstance [25].

Information regarding the common sites of EHMP may benefit the detection of high-risk individuals via early diagnosis, leading to improve cancer-survivor outcome. Therefore, we analyzed the frequency of EHPM according to tumor types. Contrary to our expectations drawn from the results of previous studies conducted in Japan [4] and Western countries [23], nasopharynx cancer ranked first in extrahepatic tumors, not gastric nor colorectal cancer. Nasopharyngeal carcinoma (NPC) is a disease with remarkable geographic distribution and a high incidence rate in South China. However, despite its prevalence, it does not rank first among extrahepatic tumors in South China [26]. The reasons for the high incidence of NPC in HCC patients are unclear. No apparent environmental [27] or shared genetic factors [28] explained the circumstance. In light of the high frequency of NPC in EHPM, head and neck screening (physical examination and flexible laryngoscopy) should be considered in those patients. These data suggest oncologists should be aware of the risk of secondary tumor development if unexpected symptoms or signs appear, as intensive follow-up may benefit early diagnosis.

A differential diagnosis of HCC should be made, as the liver being one of the most common sites of metastases. In our cohort, 30 HCC patients were diagnosed via standard clinical and imaging criteria combined with an elevated AFP level. The characteristic of HCC imaging in the MPMs was similar to that of HCC-alone patients. Dynamic CT can provide useful information for the differential diagnosis for hepatic nodules [29]. A metachronous patient presented two hepatic nodules after NPC radiotherapy in this study. CT imaging showed two nodules with distinct radiologic natures (one with typical presentation of a low-density mass in the plain CT, which changed to a high-density mass in the arterial phase with relative low density in the delayed phase of dynamic CT; the other presented metastatic cancer: enhance less intensively and washout more slowly). Postoperative pathology confirmed the case suffered hepatoma complicating hepatic metastases from NPC.

We investigated whether cancer-survivors would experience poorer prognoses after second malignant neoplasm diagnosed. Our study showed that the difference of HCC-specific survival between MPM involving HCC and HCC-alone failed to reach statistical significance. This finding was similar to that reported in several cohorts [2,23], and reflected differences in the natural course of HCC compared with most associated EHPM. HCC is always associated with a poorer survival and a more aggressive behavior than most other tumors. Several cohort studies noted that the causes of death in HCC patients with EHPM were mostly attributed to HCC itself, not to extrahepatic cancer [3,5]. In light of these data, we stressed the need to prioritize curative treatment for HCC independently of the EHPM, as the presence of an EHPM had little impact on HCC-specific prognosis. While, we noticed that no significant difference was observed in overall survival between surgery and other therapeutic options for HCC. A long-term diagnostic interval time (median 32.4 months) from the diagnosis of the first neoplasm to the subsequent tumor presumably influenced the impact efficiency of treatment options for HCC on overall survival.

A significant difference in OS between the patients in the metachronous group and the synchronous group was not surprising. Compared with metachronous patients, synchronous patients had poorer OS. As demonstrated in Table 3, synchronous and metachronous groups associated with different treatment approaches for the extrahepatic tumor, and different treatment strategies led to distinct outcomes. Thus, we suspected that treatment modality was the final influencing factor for overall survival and built a Cox multivariate model for verification. Cox analysis confirmed it and demonstrated that treatment options for extrahepatic cancer remained as the significant factor. Therefore, an increasing effort to enlarge the proportion of patients undergoing radical treatment should been taken into account, irrespective of synchronous or metachronous tumor development.

Curative resection, if possible, most effectively prolongs patient survival. However, in cases of HCC with EHPM, the choice of treatment strategy should be made carefully in conjunction with the treatment for EHPM. There are few reports on how to treat patients with MPM involving HCC, which remains a key challenge. In our cohort, hepatectomy was an independent prognostic factor associated with good outcome. In spite of the patients that had a cirrhosis background, as experienced in our center, when patients had well-preserved liver function and when there was also an absence of extrahepatic metastasis, aggressive liver resection provided an opportunity for long-term survival.

MPM patients including HCC were often treated with supportive treatment, due to the limited therapeutic options, disappointed radical surgical rate, and poor prognosis of HCC [25]. Although several papers reported MPM concomitant with surgical HCC patients [2-5,23,24], with the small number of patients, the statistical power was limited. Of noted, only two cohort studies investigated the influence of EHPM on survival with more than 20 surgical HCC [2,3]. Furthermore, both of the two papers failed to refer to HCC-specific survival, which lead to decrease the value for clinical practice. We believe the present study may provide powerful evidence to make therapeutic strategy for MPM patients with HCC, which suggest those patients should not be excluded from radical resection for HCC.

Potential limitations of our study are the long time span of the observed data and the relatively small sample size, particularly with only 35 MPM patients with resected liver tumors. It would be more powerful the results from multicenter studies with larger numbers, considering the different geographic distribution of HCC. Nonetheless, the consistency of our findings with previous clinical surveys reinforces the validity of our conclusions.

## Conclusions

In conclusion, NPC, colorectal, and lung cancers were the tumors most frequently accompanying HCC. Careful follow-up and active treatment is suggested for these patients. Curative treatment should been taken into account both for synchronous and metachronous patients. The efficiency of the treatment against HCC and EHPM had great influence on the prognosis. Moreover, EHPM did not confer a poorer HCC-specific survival, and those patients should not be excluded from curative therapies.

## Abbreviations

MPM, Multiple primary malignancies; EHPM, Extrahepatic primary malignancies; HCC, Hepatocellular carcinoma; OS, Overall survival; RFS, Recurrence-free survival; HBsAg, Hepatitis B surface antigen; AFP, Alpha-fetoprotein..

## Competing interests

The authors declare that they have no competing interests.

## Authors’ contributions

QAZ, JLQ, RHZ, YJL, SPL and YFY have made substantial contributions to conception and design of the study. QAZ, JLQ, PZH, RHZ and YZ carried out acquisition of data. JLQ, YJL, BKL and JH carried out analysis and interpretation of data. JLQ, YFY, and XML have been involved in drafting the manuscript. All authors read and approved the final manuscript.

## Pre-publication history

The pre-publication history for this paper can be accessed here:

http://www.biomedcentral.com/1471-2407/12/148/prepub
